# First person – Claudia L. Charles-Niño

**DOI:** 10.1242/dmm.052366

**Published:** 2025-03-25

## Abstract

First Person is a series of interviews with the first authors of a selection of papers published in Disease Models & Mechanisms, helping researchers promote themselves alongside their papers. Claudia L. Charles-Niño is first author on ‘ Reduced growth and biofilm formation at high temperatures contribute to *Cryptococcus deneoformans* dermatotropism’, published in DMM. Claudia is a postdoctoral associate in the lab of Dr Luis R. Martinez at University of Florida College of Dentistry, Gainesville, FL, USA, investigating medical mycology, pathogenesis and immunology, focusing on understanding the molecular interactions between pathogens and host.


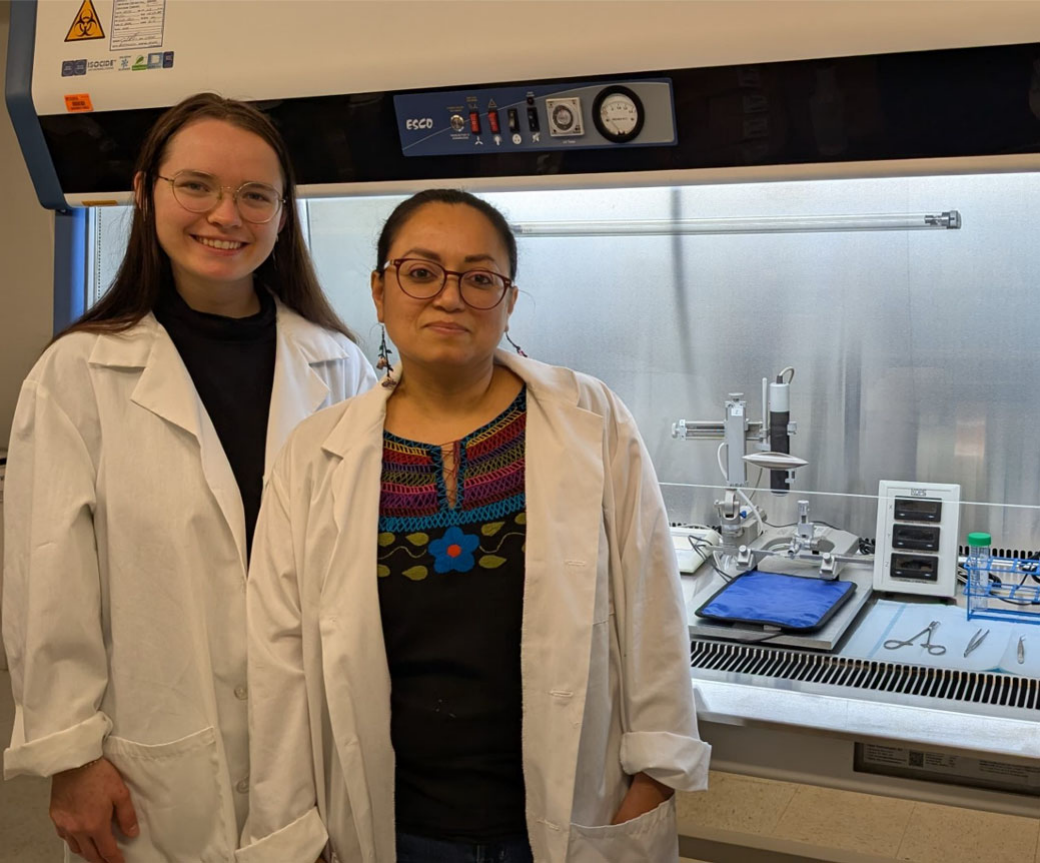


**Claudia L. Charles-Niño (right) pictured with co-author Melissa Munzen (left)**

**Who or what inspired you to become a scientist?**

I became a scientist because, as a child, I was inspired by the movie ‘Outbreak’ and wanted to be like Dr Lisa Aronson. The film, which depicts scientists racing to contain a deadly virus, fascinated me and sparked my passion for understanding infectious diseases. I was so determined to become an infectious diseases researcher that I even considered joining the army in my home country, Mexico, but I was too short to qualify. Instead, I researched other ways to become a scientist and learned that I needed to complete a bachelor's degree followed by graduate studies. From that moment on, I planned my career with this goal in mind. My interest in microbiology and disease-causing microorganisms grew stronger, leading me to focus my research on infectious diseases. I started working in a lab at 18, and, ever since, I have been dedicated to studying how pathogens interact with their hosts and cause disease.

**What is the main question or challenge in disease biology you are addressing in this paper? How did you go about investigating your question or challenge?**

Our study addresses why *Cryptococcus deneoformans* has a stronger association with skin infections compared to *Cryptococcus neoformans*, which is more commonly linked to systemic infections. We hypothesized that differences in thermotolerance and biofilm formation influence *C. deneoformans* dermatotropism. To investigate this, we used a murine wound model to compare fungal persistence, biofilm formation and immune responses. We also analysed thermotolerance across multiple strains and examined the expression of heat-shock proteins (HSP60 and HSP70) at different temperatures. Our findings suggest that *C. deneoformans* is less thermotolerant than *C. neoformans*, which may explain its preference for cooler tissues like the skin.

**How would you explain the main findings of your paper to non-scientific family and friends?**

Think of people and their climate preferences – some thrive in warm, tropical weather, while others prefer more temperate conditions. *Cryptococcus neoformans* is like someone who enjoys the heat, adapting well to the body's high temperature (around 37-39°C) and spreading to deeper organs like the brain and lungs. *Cryptococcus deneoformans*, on the other hand, struggles with high temperatures and instead settles in the skin, where the temperature is lower (around 32-34°C). To survive, *Cryptococcus* doesn't exist alone – it forms communities of microbial cells that stick together and produce a protective barrier, known as a biofilm. This is similar to how people in difficult environments band together and build shelters to protect themselves from harsh conditions. We found that *C. deneoformans* needs to build more complex shelters to protect itself from body temperatures that are higher than its natural niche (around 30°C) in comparison with *C. neoformans*. Our research helps explain why these two fungi behave differently and how temperature plays a key role in fungal infections.

[…] our study highlights how fungal adaptation to temperature influences disease progression, which is particularly relevant as global temperatures rise.

**What are the potential implications of these results for disease biology and the possible impact on patients?**

Our findings provide insight into why *C. deneoformans* prefers to infect the skin, while *C. neoformans* is more likely to cause systemic disease. This knowledge can help clinicians recognize risk factors for skin infections and improve diagnostic strategies. Additionally, our study highlights how fungal adaptation to temperature influences disease progression, which is particularly relevant as global temperatures rise. Understanding these mechanisms may lead to targeted antifungal treatments that disrupt biofilm formation or exploit fungal thermosensitivity, ultimately improving patient outcomes.

**Figure:**
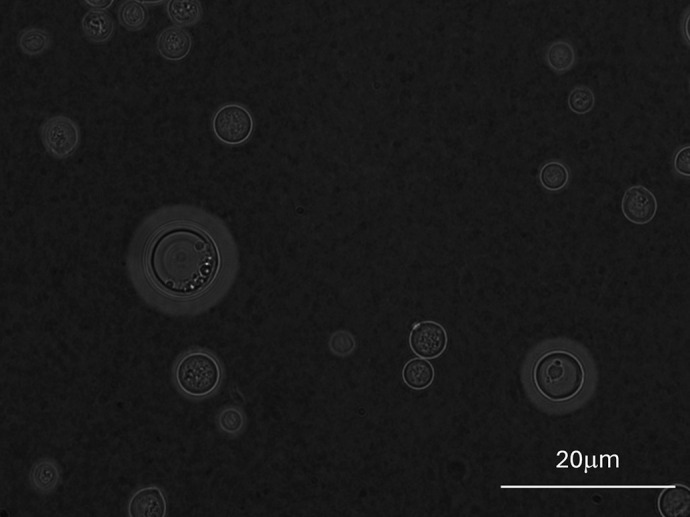
India ink staining of *Cryptococcus neoformans*, showing its complex capsule – a key virulence factor.

**Why did you choose DMM for your paper?**

I chose DMM to publish this paper because its scope aligns perfectly with our research on the mechanisms underlying *C. deneoformans* infection and its thermal susceptibility. DMM is a well-regarded journal that focuses on translational disease research using model systems, making it an ideal platform to highlight our findings on how thermotolerance influences fungal dermatotropism and pathogenesis. The journal's emphasis on host–pathogen interactions and disease mechanisms ensures that our work reaches an audience interested in infectious diseases and immunology. Additionally, DMM provides Open Access options, making our findings widely accessible to researchers, clinicians and public health professionals. While publication fees were also a consideration, the journal's balance of quality, visibility and affordability made it the best fit for disseminating our research.

**Given your current role, what challenges do you face and what changes could improve the professional lives of other scientists in this role?**

As a postdoctoral researcher and lab manager, one of the biggest challenges I face is balancing research responsibilities with administrative and mentoring duties. Managing lab operations, training students and ensuring compliance with safety protocols often compete with time dedicated to experiments and data analysis. Additionally, securing funding is a constant struggle, as early-career scientists often have limited opportunities to apply for grants independently. This challenge is even greater for US non-citizen researchers, who may face additional restrictions on grant eligibility and fewer funding opportunities. Expanding funding options for international scientists and providing clearer pathways to long-term research positions would greatly benefit the scientific community.

[…] a strong, dedicated researcher working alongside a PI can be just as impactful as one leading a lab.

**What's next for you?**

I recently applied for a research assistant professor position, as I want to continue my career in academia. My goal is to remain actively involved in research while securing my own funding, but I prefer not to take on the role of a principal investigator (PI). While I have the capability to be a PI, I find greater fulfilment in contributing to research under the leadership of an established investigator. Just like in an orchestra, where the second violin plays a crucial role in creating harmony, I believe that a strong, dedicated researcher working alongside a PI can be just as impactful as one leading a lab. I thrive in an environment where collaboration and mutual respect drive scientific discovery, and I value the professional dynamic I have with my current mentor. Moving forward, I aim to continue making meaningful contributions to research while maintaining the flexibility and focus that my role allows.

**Tell us something interesting about yourself that wouldn't be on your CV**

I recently discovered a passion for cooking, mostly because I miss the flavours of Mexico. I cook every chance I get, but I enjoy it even more when I can share a meal with good company. Recently, I started growing my own vegetables and fruits in my garden. It's still a work in progress. My love for movies is also a big part of who I am. My idea of a perfect day off would start with spending time in my garden, then preparing a homemade meal with ingredients harvested from my own backyard and, finally, unwinding with a good movie like ‘Contagion’, which, of course, perfectly aligns with my passion for infectious disease research!
